# Physical activity during pregnancy: a systematic review for the assessment of current evidence with future recommendations

**DOI:** 10.1186/s13102-022-00524-z

**Published:** 2022-07-16

**Authors:** Leona Cilar Budler, Marko Budler

**Affiliations:** 1 Faculty of Health Sciences, University of Maribor, Zitna ulica 15, 2000 Maribor, Slovenia; 2 School of Economics and Business, University of Ljubljana, Kardeljeva ploscad 17, 1000 Ljubljana, Slovenia; 3 Fitness Association of Slovenia, Cesta 24. junija 23, 1231 Ljubljana, Slovenia

**Keywords:** Exercise, Intervention, Sport, Lifestyle, Health outcome

## Abstract

**Background:**

Physical activity is essential to maternal and infant health. Healthcare professionals should inform pregnant women about benefits of physical activity to prevent possible health issues. Those recommendations should elaborate on relevant contemporary evidence. The aim of this study was to review evidence-based recommendations for physical activity during pregnancy.

**Methods:**

A systematic search, analysis and synthesis of conducted randomised controlled trials (RCTs) was conducted from October 2021 to June 2022 in following databases: PubMed, CINAHL, ScienceDirect and Web of Science. Literature was searched using inclusion and exclusion criteria and following PRISMA recommendations.

**Results:**

Benefits for pregnant-women health and well-being were reported while performing aerobic exercise, lumbar stabilization and stretching exercise, water exercise, nerve and tendon-slip exercise, resistance training and strength training. For all exercise modalities it is recommended to perform moderate intensity activities during the whole time of pregnancy.

**Conclusions:**

This systematic literature review supplements current knowledge on physical activity of pregnant women. Exercise interventions are listed and suggested in an integrative model with physical-fitness components to contextualize and promote physical activity among pregnant women.

**Supplementary Information:**

The online version contains supplementary material available at 10.1186/s13102-022-00524-z.

## Background

Physical activity (PA) is defined as “bodily movement produced by skeletal muscles that results in energy expenditure” [1]. PA is believed to be essential to healthy pregnancy. Historically, Biblical writers noticed that Hebrew slave women gave birth more easily than sedentary Egyptian mistresses [2]. Moreover, it is believed that PA during pregnancy limits gestational weight-gain [3–5], decreases risk of maternal mental disorders after childbirth [6, 7] and improves body image satisfaction [8]. PA in pregnancy is pivotal to facilitating positive health outcomes in infants [9].

To corroborate the role of PA in pregnancy and the expected favourable health outcomes for pregnant women and infants, this study distinguishes between the terms PA, exercise (intervention), and physical fitness that are all distinct concepts. However, these concepts (terms) are often used interchangeably. In line with a seminal paper, we deem exercise as “a subset of PA that is planned, structured, and repetitive and has as a final or an intermediate objective the improvement or maintenance of physical fitness”. In addition, physical fitness is deemed “a set of attributes that are either health- or skill-related” [10].

Pregnant women tend to demonstrate a lack of knowledge regarding PA during pregnancy [9, 11, 12]. The reasons for insufficient knowledge include but are not limited to the mothers’ race [13], socio-economic and cultural context [14], and maternal education [15]. Relatively low degree of pregnant women report that they had received prescription in terms of exercise interventions from health providers during pregnancy [16]. In addition, past research identified an important barrier to enhancing pregnant women’ knowledge about PA—the absence of PA-related domains in the development of professional healthcare professionals [17]. Due to lack of guidance, pregnant women access information about healthy lifestyle during pregnancy via the internet with questionable credibility [18, 19].

Mottola et al. who performed a literature review and identified some benefits of prenatal PA, provided preliminary guidance for pregnant women and healthcare professionals on prenatal PAity [20]. Unsurprisingly, the women who were given guidelines for PA during pregnancy reported exercising [21].

In addition to potential issues with credible sources of information and lack of novel guidance, healthcare professionals need multiple sources to develop an “integrative approach” for promotion of PA to pregnant women [22, 23]. From the standpoint of the authors,an “integrative approach” should, for instance, account for tailored exercise interventions that could be useful to pregnant women with specific goald, and facilitate the adherence of pregnant women to PA [24, 25].

The aim of the current study is hence two-fold. First, we respond to more-recent calls for evidence-based recommendations that would enhance promotion of PA during pregnancy (see e.g., [26]). We have listed and contextualized the recommendations to be provided to pregnant women, professional healthcare professionals involved in nursing (e.g., midwives), and wider society. Further, by identifying exercise modalities, examining characteristics of exercise interventions with a systematic review, and corresponding health outcomes we address a recent call from DiPietro et al. [27] to advance current knowledge and empower healthcare professionals. As interventions should be tailored (e.g., goal-oriented to a physical-fitness components), our second aim is to characterize the interventions from main findings, and further elaborate characteristics such as exercise modality. By corroborating the exercise interventions for pregnant women, we can enhance the adherence to PA and improve the promotion of PA among the healthcare professionals. Ultimately, the current study concludes with the contributions to the learning system and an identification of intricate contemporary challenges to be addressed in the future research.

## Methods

### Design

A systematic review is a summary of the medical literature that uses explicit and reproducible methods to systematically search, critically appraise, and synthesize a specific health issue [28]. Following steps were taken into account when performing a systematic review: (1) defining research question; (2) preliminary literature search; (3) development of search string, inclusion and exclusion criteria; (4) literature search and analysis; (5) literature synthesis; (6) assessment of literature quality and bias; and (7) interpretation of findings and proposition of future directions. When performing a systematic review, the Additional file 1: PRISMA guidelines were followed [25] (Additional file 1: Appendix 1).

### Search methods

For the development of research question, a Population, Intervention and Outcome—PICO [29] format was used. The research question was: *How do exercise interventions improve the health of physically-active pregnant women and their infants?* Exclusion and inclusion criteria were developed based on the preliminary literature search and PICO research question (Table 1).

**Table 1:** Search criteria and inclusion and exclusion criteria

Databases	PubMed, CINAHL, ScienceDirect and Web of Science	
	Inclusion criteria	Exclusion criteria
Limits	English language, Publication between June 2017 and June 2022, RCTs	Other languages Publication before 2017 Pilot studies, protocols, secondary analyses, commentaries, cohort studies
Population (P)	Pregnant women (1.–37./42. week)	Postpartum women, children, infants
Intervention (I)	PA (e.g., cardiorespiratory, fitness, endurance, strength, etc.)	Interventions that did not involve exercises and span beyond PA (e.g., mindfulness, diet)
Comparison (C)	Pregnant women (1.–37./42. week) without intervention (placebo groups)	–
Outcome (O)	On pregnant women during pregnancy and post labour (hormones, weight, gestational diabetes, pain, mental well-being, quality of life) and, if applicable, on infant—denoting something in an early stage of its development (development, growth, feelings)	Not related to pregnant women’s, children’s health outcomes or exercise modality

We searched for literature published between June 2017 and June 2022, in English language and RCTs. As described by Hiebl [30], authors can limit their literature search by publication time. However, reasons should be justified and mentioned in the limitation section. The aim of this systematic review is to get the newest and best evidence in this field, thus, only literature published in last five years were examined. We provide up-to-date evidence which is often neglected by authors of overviews [31]. We included only RCTs because they are assessed as the most credible in the hierarchy of evidence [23]. Other studies were excluded (Additional file 1).

A search string was developed based on preliminary literature search and was taken into account in the process of literature search (Table 2).

**Table 2:** Search results

Search string	Database	Number of hits
(sport* OR exercise* OR physical activit* OR fitness OR aerobic OR training*) AND (pregnant* OR pregnancy OR gestation* OR gestate OR gestational OR maternity OR maternal OR prenatal)	PubMed	740
	CINAHL	282
	Web of Science	856
	ScienceDirect	214

* substitutes for a string of characters (e.g., physical activit* can stand for physical activity or physical activities)

### Search outcomes

**Fig. 1:**
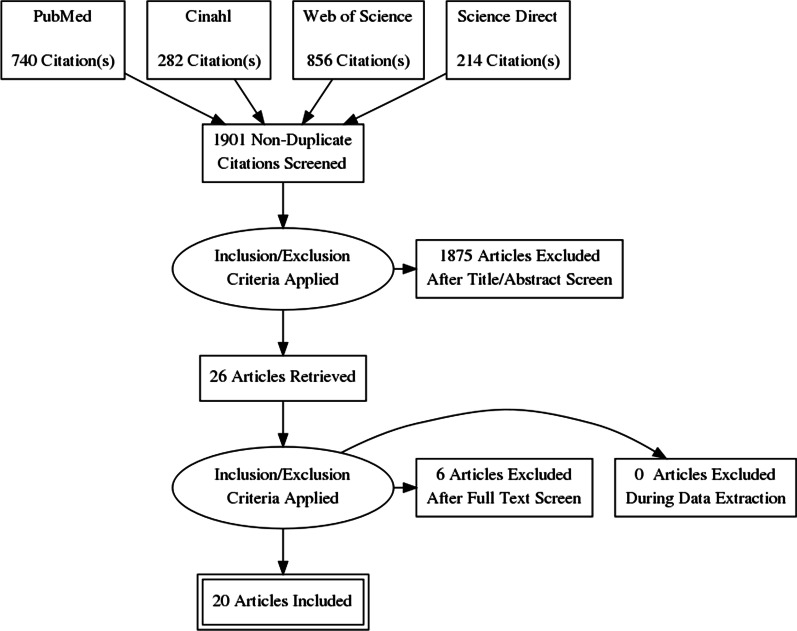
PRISMA flow diagram

Using the developed search string 2092 records were identified in four databases. After duplicates exclusion, literature was checked by title and abstract; 26 articles were retrieved and checked by the full text. Finally, 20 articles were included in the final analysis and synthesis. Steps of literature search are presented in Fig. 1 [32].

### Quality appraisal

The data quality was assessed using the Grading of Recommendations, Assessment, Development, and Evaluation (GRADE) system [33, 34]. GRADE identified its five categories—study limitations, imprecision, inconsistency, indirectness, and publication bias. GRADE quality level is interpreted as high (++++), moderate (+++), low (++), or very low (+).

### Data extraction

Data were extracted using standardized data form in Microsoft Excel® by two reviewers. The first reviewer exported data and second reviewer checked for data accuracy. The literature screening was carried out independently by two researchers. Disagreements were solved by consensus. We extracted study characteristics such as study sample, exercise intervention, main study results and conclusions.

### Data synthesis

A narrative synthesis was conducted for all included studies. Results were synthesized by PA modality and intensity or duration of PA. Also, results were synthesised due to the intervention effectiveness.

## Results

In total, 20 articles were included in the final analysis (Table 3). Studies which fit the inclusion criteria were analysed due to sample, intervention, results and conclusions. Excluded studies are listed in the Additional file 2: Appendix 2.

**Table 3:** Study characteristics

No.	Reference	Sample age M (± SD)	Intervention	Results	Outcomes	
					Primary	Secondary
1	[35]	456 pregnant women EG 31.75 (4.68) CG 31.04 (3.78)	Moderate aerobic exercise performed three days per week (50–55 min per session) for 8–10 weeks to 38–39 weeks of gestation	The prevalence of gestational diabetes was significantly higher in the control group (*p* = 0.033)	Total maternal weight gain (kg) and excessive gestational weight gain	Maternal gestational age at delivery, type of delivery and birth weight
2	[36]	20 pregnant women with low back pain between 19 and 29 weeks of gestation Stabilization group 30(6) Stretching group 29(6)	(1) Lumbar stabilization exercise (2) Stretching exercise	Both interventions showed significant improvement in postural stability the velocity sway parameter, and significantly increased activation (*p* > 0.05) of the external abdominal oblique muscle after intervention	Pain, disabilities, postural control and muscle activation	–
3	[37]	639 pregnant women between 16 and 20 weeks of gestation IG 27.2 (5.5) IG Missing 26.0 (4.9) CG 27.3 (5.5) CG Missing 25.8 (5.8)	16-week supervised exercise program including aerobic and resistance training delivered in 60-min sessions 3 times per week	There was no significant difference for postpartum depression between groups	Preterm birth and preeclampsia	Maternal and child health outcomes
4	[3]	436 pregnant women at < 20 weeks of gestation UC 31.8 (5.6) HE + PA 31.9 (5.3) HE 32.5 (5.5) PA 31.7 (5.1)	5 face-to-face and ≤ 4 telephone coaching sessions using the principles of motivational interviewing	In the intervention group, women achieved less gestational weight gain. No improvements were seen in fasting or post load glucose levels or insulin concentrations	Gestational weight gain and homeostasis model assessment insulin resistance at 24–28 weeks	Physical activity, nutrition, glucose concentrations 1 and 2 h after glucose ingestion, fasting insulin concentrations, insulin levels at 1 and 2 h after glucose ingestion, GDM, birth weight, gestational age, and small-for-gestational age (SGA) or large-for-gestational age (LGA) infants
5	[38]	129 pregnant women from 20 to 37 week of pregnancy EG 32.12 (4.43) CG 30.58 (4.75)	The SWEP (study of water exercise during pregnancy) method	The health-related quality of life score decreased significantly between weeks 12 and 35 of gestation, except for the mental health component	Quality of life	**–**
6	[13]	594 pregnant women EG 27.2 (5.3) CG 27.1 (5.7)	The exercise-based intervention conducted three times/week for 16 weeks from 16–20 to 32–36 weeks' gestation	There were no significant differences in the incidence of preterm birth, pre-eclampsia, gestational weight gain, gestational diabetes, birth weight, infant length, and head circumference between groups	Preterm birth and pre-eclampsia	Gestational weight gain, gestational diabetes mellitus (GDM), birth weight, small and large-for-gestational age, infant length, and head circumference
7	[39]	645 pregnant women Lifestyle Advice 31.60 (4.63) Standard Care 31.45 (4.63)	An intervention consisting of six sessions (three face-to-face, two provided by the dietitian after trial entry and at 28 weeks’ gestation, one provided by a research assistant at 36 weeks’ gestation); three telephone calls at 20-, 24- and 32-weeks’ gestation	There was no statistically significant difference in the proportion of infants with birth weight above 4.0 kg between groups (*p* = 0.732)	Proportion of infants with birth weight > 4 kg	Preterm birth; perinatal mortality; infant birth weight; hypoglycaemia; admission to neonatal intensive care unit or special care baby unit; hyperbilirubinaemia; nerve palsy; fracture; birth trauma; shoulder dystocia; corticosteroid use; respiratory distress syndrome; discharge home on oxygen; patent ductus arteriosus; proven systemic infection; retinopathy of prematurity; necrotising enterocolitis; neonatal encephalopathy; seizures; length of hospital stay; and infant not exclusively breast fed at hospital discharge
8	[40]	120 pregnant women 31.8 (5)	A supervised physical conditioning program consisting of three 60-min sessions per week for the whole duration of pregnancy (weeks 9–38)	No differences were found between the groups in maternal weight. The proportion of women with weight loss ≥ 9 kg at 6 weeks postpartum was higher in the exercise compared with the control group (*p* = 0.02). The ductus arteriosus pulsatility index at 20 weeks (*p* < 0.05) and the ejection fraction at 36 weeks (*p* < 0.05) were higher in the exercise compared with the control group	Maternal weight gain during pregnancy	Fetal cardiac function parameters evaluated at 20, 28 and 36 weeks' gestation, Cesarean section, preterm delivery, induction of labor and birth weight
9	[41]	33 pregnant women IG 28.4 (3.62) CG 28.8 (4.67)	Nerve and tendon-slip exercise	There were no significant differences between the groups in symptoms and clinical tests (*p* > 0.05). Patients receiving treatment showed a decrease in symptom severity and functional capacity, but only the former showed a significant decrease in group 2 (*p* > 0.05)	Effectiveness of home exercise in pregnant women with carpal tunnel syndrome	–
10	[42]	134 pregnant women in second trimester Resistance Exercise 28 (5) Pregnancy Education 29 (4) Wait List 29 (4)	12 weeks of wait list, education classes, or twice weekly low-to-moderate intensity resistance training	Scores were unchanged after resistance training but significantly decreased for the education (*p* = 0.001) and wait list (*p* < 0.001) groups, whereas post-test vitality scores for the pregnancy group were significantly higher than the wait list (*p* = 0.05) but not the education group (*p* = 0.27)	Fatigue-related domains of quality of life and mood	–
11	[43]	91 pregnant women IG 31.3 (3.8) CG 31.4 (4.7)	3 weekly supervised exercise sessions (35 min of moderate intensity walking/running and 25 min of resistance training), until delivery	There was no between-group difference in depression (*p* = 0.55)	Psychological wellbeing	Postpartum depression
12	[44]	516 pregnant women IG 31.4 (4.3) CG 30.6 (4.1)	Unsupervised water exercise twice a week for a period of 12 weeks	Low back pain intensity was significantly lower in the water exercise group (*p* = 0.04). More women in the water exercise group reported no low back pain at 32 weeks (*p* = 0.07)	Low back pain intensity	Days spent on sick leave, disability due to low back pain and general health
13	[4]	435 pregnant women Usual Care 31.8 (5.6) HE + PA 31.9 (5.3) HE 32.5 (5.5) PA 31.7 (5.1)	Healthy eating and PA promotion intervention, a healthy eating promotion intervention, or a PA promotion intervention	Between-group total cost and effect differences were not significant, besides significantly less gestational weight gain	Gestational weight gain, fasting glucose, insulin resistance, quality adjusted life years, and societal costs	–
14	[45]	724 pregnant women IG 30.5 (4.4) CG 30.4 (4.3)	12-week standardized exercise program, including both aerobic and strength training (20–36 weeks′ gestation)	No between-group difference in serum 25(OH)D and related parameters was identified	Gestational diabetes	Effects on parathyroid hormone, total and corrected calcium, magnesium, phosphate, and Vitamin D-binding protein
15	[46]	241 pregnant women (n = 122) IG 31.6 (3.9) CG 31.3 (4.3)	Exercise program (walking goal of 10,000 steps/day)	The intervention group achieved higher protein intake and healthy diet scores (*p* < 0.005) but step counts were similar averaging 6335 steps/day. Pregnancy and infant outcomes were similar between groups	Proportion of women who achieved gestational weight gain	–
16	[47]	639 pregnant women IG 27.7 (5.6) CG 27.7 (5.7)	16-week exercise program starting between the 16th and 20th weeks of gestation, 3 times a week, lasting 60 min per session	Children from women in the exercise group had higher language score at age 2 years and higher cognitive score (standardized at age 4 years. No effects of exercise during pregnancy were observed in the motor domain at 1, 2, and 4 years	Preterm birth and preeclampsia	–
17	[48]	150 pregnant women IG 32.4 (5.4) CG 33.4 (4.7)	10,000 steps a day, equivalent to 30 min per day of moderate physical activity over the week (≥ 5 days)	During the 35 and 37 gestational weeks, women in the intervention group had higher mean PA than women in the control group (*p* = 0.01)	Maternal and perinatal outcomes	–
18	[49]	90 pregnant women IG 29.46 (5.78) CG 28.94 (5.33)	Virtual group in a social network (What's app) received individually diets and materials about that how to track its effect on their weight gain during pregnancy	There was a statistically significant difference at the end of intervention in the level of daily physical activity (*p* < 0.001)	Amount of changes in the mean daily total physical activity level	Measuring the level of changes in other subgroups of daily physical activity alongside with the level of weight gain during pregnancy
19	[50]	305 pregnant women	6-month digital lifestyle intervention (the HealthyMoms app)	Digital lifestyle intervention is effective in promotion of healthy dietary habits in women representing all BMI-categories and decrease weight gain during pregnancy in women with overweight and obesity without compromising offspring growth	Healthy weight gain, diet, and physical activity	Body fatness, dietary habits, moderate-to-vigorous physical activity, glycemia, and insulin resistance
20	[51]	54 pregnant women n 20 to 26 weeks of gestation	Water Exercise in Pregnancy program	Level of discomfort and musculoskeletal complications in the intervention group was significantly reduced compared to the control group (*p* < 0.001)	Musculoskeletal pain	–

*PA* physical activity, *p* *p* values, statistical significance, *SWEP* study of water exercise during pregnancy, *n* number of participants, *BMI* body mass index, *GDM* gestational diabetes mellitus, *M* mean value, *SD* standard deviation, *IG* intervention group, *CG* control group, *EG* exercise intervention group, *HE* healthy eating, *PA* physical activity, *HE + PA* both HE and PA, *UC* usual care

Studies included in the analyses involved various number of participants. Those numbers varied between minimum of 20 [27] and maximum of 1023 pregnant women [38]. All studies had intervention and control group. However, all studies did not report mean and standard deviation for each group. Interventions were the following: moderate aerobic exercise, lumbar stabilization and stretching exercise, resistance training, water exercise, various moderate-intensity exercises, physical conditioning program, cycling program, nerve and tendon-slip exercise, and individual or group sessions with a personal trainer. Studies outcomes are assessed as positive or negative for pregnant women or infant in the Table 4. Details about each intervention are provided in the Table 5. In the remainder of this study the observed studies are assessed for quality using the GRADE system (table in Additional file 3: Appendix 3). All studies included were RCTs because of the search criteria. RCTs are seen as a high-quality body-of-knowledge [34]. When assessing quality of each evidence, study design, study limitations, inconsistency, indirectness, imprecision and risk of bias were considered. The results of the assessment are as follows. Among 18 units-of-analysis, three were assessed as moderate quality, eight as low quality, and seven as very low quality. In these seven, quality scored was lower due to numerous limitations (e.g., the absence of the control group, small study sample, lack of blinding, lack of robust analyses, etc.), higher deviations in CIs for interventions, or risk of bias.

**Table 4:** Data synthesis by intervention and results

No.	Exercise interventions	Results
1	Moderate aerobic exercise	Positive
2	Lumbar stabilization and stretching exercise	Positive
3	Moderate aerobic and resistance training	No changes
4	Healthy lifestyle intervention (aerobic and resistance PA)	Positive
5	Water exercise	Positive
6	Various moderate-intensity exercises	No changes
7	PA plan	No changes
8	Physical conditioning program	Positive
9	Cycling program	No changes
10	Nerve and tendon-slip exercise	Positive
11	Moderate intensity walking/running and resistance training	No changes
12	Water exercise	Positive
13	PA	Positive
14	Aerobic and strength training	No changes
15	Exercise program (walking)	No changes
16	Aerobic activities, strength training and floor exercises	No changes
17	Moderate physical activity (walking)	No changes
18	Educational intervention (social network)	Positive
19	Digital lifestyle intervention (the HealthyMoms app)	Positive
20	Water Exercise	Positive

*PA* physical activity

**Table 5:** An in-merge of evidence-based exercise interventions by the exercise modality

Exercise modality	Exercise intervention	Expected positive results
Strengthening	Low-to-moderate intensity resistance exercise twice per week for 12 weeks and, depending on availability (dual leg extension, dual leg press, dual arm lat pull, dual leg curl, lumbar extensions and a standing abdominal exercise) [42] Dumbbells, machines, or elastic bands [47]	Adverse changes in symptoms of energy and fatigue
Balance	Two static upright balance postural tasks: two-legged stance either with eyes open and with eyes closed Three balance sitting tasks on a Swiss ball: to remain seated on the ball in a static position, with both feet resting on the floor and hands resting on the sternum; sitting on the ball, raise the lower right leg off the floor and hold the lift for 10 s, with hands resting on the thighs; sitting on the ball, raise the lower left leg off the floor and hold the lift for 10 s, with hands resting on the [36]	Pain reduction; improved balance performance
Stretching	The tendon slip exercises (flexion, flat, hook, punch, table-top and flat-punch) [41] Nerve-gliding exercises (moving the fingers and wrists in six different positions, focussing on the median nerve consisting of the disease grip, finger lengthening, wrist extension, thumb extension, forearm supination and gentle gait) [41]	Decrease in carpal tunnel syndrome severity and functional capacity
Aerobic exercise	General aerobic and resistance training [3, 47]	Lower gestational weight gain and sedentary behaviour; greater moderate-to-vigorous PA
	Gradual warm-up; aerobic exercises; light muscle strengthening; coordination and balance exercises; stretching exercises; pelvic floor strengthening; relaxation and final talk [35]	Lower maternal weight gain; better OGTT results; lower chance to get GDM; lower ratio of macrosomia of neonate
Exercise modalities combined	Moderate PA in water [51]: warm-up, main phase (with an aerobic element, followed by strength and endurance exercises) and final stretching and relaxation [38]	Effective in reducing musculoskeletal complications Better perineum status and physical functioning; lower pain; better general health, vitality, social functioning, role emotional and physical
	Physical conditioning program:10 min of warming up, 25 min of cardiovascular exercise, 10 min of strengthening exercises, 5 min of coordination and balance exercises, 5 min of pelvic floor exercises and 5 min of stretching and relaxation [40]	Faster postpartum weight loss; higher ductus arteriosus pulsatility index and the ejection fraction
	Water exercise: four swimming laps (100 m in total), six AquaMama exercises and four laps [44]	Lower back pain intensity
	Moderate PA, reducing sedentary time, upper and lower limb resistance exercise, increasing number of steps per day, increasing activity during weekends [4]	Lower gestational weight gain; more costly and effective intervention

*PA* physical activity, *OGTT* oral glucose tolerance test, *GDM* gestational diabetes mellitus

Out of 20 identified RCTs, 11 (55.00%) reported positive results of implemented intervention on maternal or infant health outcomes (Table 4). Others showed no changes or did not report the result.

To help professional healthcare professionals in promoting PA we further categorize interventions by exercise modality (Table 5). Among a range of exercise modalities, four exercise modalities, namely strengthening, stretching, balance, and aerobic exercises, are commonly found in the existing body of literature that focuses on positive results of physical activity interventions on health outcomes [52–55].

Table 5 categorizes main findings with respect to exercise modality and their expected positive results on pregnant-women health and well-being. The favourable health outcomes of performing moderate aerobic exercise were most extensively examined [35, 45], followed by strengthening see e.g., [42], or a hybrid form using both [38, 44]. For aerobic exercise, treadmill, walking, and other aerobic-exercise programs are advised (see Table 5). A more comprehensive hybrid form of PA program included moderate aerobic exercise with gradual warm-up; aerobics; light muscle strengthening; balance; stretching; strengthening; and relaxation with final talk [35]. In addition, [56] proposed an exercise program of both aerobic and strength training among Norwegian pregnant women to examine vitamin-D mediated effect on maternal and fetal health outcomes.

Some of the remaining studies also focused on specific sub-types of, for instance, stretching [36], and aerobic exercise [47]. In fact, practitioners are advised to suggest pregnant women the tendon slip exercises and the nerve-gliding exercises. Gestational diabetes mellitus was the main research subject by [4]. Authors revealed that, complementary to healthy eating, exercise limits the gestational weight gain. What is more, their findings pinpoint to a comprehensive approach, comprised of, for instance, moderate PA, reducing sedentary time, and strengthening.

Fontana Carvalho et al. [36] reported that involved pregnant women were included in either lumbal stabilization exercise group or lower limb and trunk stretching exercise group. Both interventions showed positive results in pain reduction caused by or perceived as a result of pregnancy. Rodríguez-Blanque et al. [38] reported that pregnant women performed a moderate PA consisting of a warm-up; aerobic exercise, strengthening, and stretching with relaxation to limit the negative effects on the body and to optimise well-being, mood and sleep patterns. Finally, positive results were also seen by combing individual diet and PA [39]. Moreover, PA intervention entailed positive results in maternal diet quality. Similarly, [40] proposed a supervised PA program consisting of warm-up, aerobic exercise, strengthening, balance, and stretching with relaxation. Hereby, intensity of PA was mild-to-moderate. The PA showed positive results in weight loss at 6 weeks post-partum.

Among the combined exercise modalities, [35] introduced a 10-min warm-up (walking and stretching) with a main section that lasted 30–35 min and included moderate intensity aerobic and resistance exercises. Activity ended with a cool down (walking, stretching, relaxation and pelvic floor muscle training). Gustafsson et al. [45] proposed a 12-week standardized PA program where women were encouraged to perform exercise modalities at home at least twice a week. Fontana Carvalho et al. [36] reported success of exercise interventions with a combination of stabilization of lower limbs and stretching. With an aim of reducing carpal-tunnel symptoms, pregnant women also conducted tailored nerve and tendon slip exercises on daily basis [41]. With respect to duration, [44] suggested water exercise to be followed for 12 weeks. Finally, a PA program aimed at conditioning was developed to encourage pregnant women to perform exercises throughout the entire pregnancy [40].

## Discussion

Our findings reveal that past research on PA in pregnancy focused mostly on health outcomes for the pregnant women and infants. Similar to Evenson et al. (2014), our analysis revealed heterogeneity of findings in terms of exercise modality, duration, and intensity. To overcome some of these shortcomings, we call for a standardization in terms of measurable and comparative characteristics of PA in pregnancy. Following this train-of-thought, a commonly used FITT framework (see e.g., [57]) has been applied to numerous sub-domains where exercise prescriptions from the healthcare professionals play a significant role, ranging from patients with cancer [58], exercise prescriptions for cardiometabolic health [59], and occasionally as a one-size-fits-all to general population [60].

Applying a framework similar to FITT to pregnant women would allow for quantifying the common characteristics of PA in pregnancy (e.g., intensity). The development of such framework that would guide the hands-on recommendations from the healthcare professionals would require acknowledging for any challenges pregnant women might have as a result of, for instance, deteriorating health, lack of physical fit, and maturity of pregnancy [61, 62]. Finally, a growing number of pregnant women have a propensity to remain physically fit notwithstanding pregnancy. Physical fitness is defined as a»state characterized by: (a) an ability to perform daily activities with vigour; and (b) demonstration of traits and capacities that are associated with low risk of premature development of physical inactivity [63]. In a broader sense, one’s physical fitness represents their capability to carry out a range of exercise modalities and daily tasks. Campbell et al. [64] emphasize the need to improve this capability concomitantly with the management any fatigue, stress, or change in health condition which is especially relevant assertion to the pregnant women. Moreover, there is a need for development of efficient physical activity interventions for pregnant women. Marini et al. [65] proposed a study protocol to design physical activity intervention for pregnant women to include in childbirth preparation classes evaluating its feasibility and efficacy on quality of life, PA levels and other outcomes.

The physical fitness is achieved, maintained and facilitated by prescribing exercise that accounts for the components of physical fitness. These components might differ with respect to the existing literature; however, most commonly the components are cardiorespiratory fitness [66], muscular strength [67], muscular endurance [68], body composition [69], and flexibility [55, 70]. Elaborating on the physical-fitness components could reveal complementary outcomes (in addition to health outcomes identified by the current study and past research) relevant to pregnant women that aim to advance their PA. We suggest the future research to focus on further examination of the complementary role of maintaining physical fitness, to account for the associated health- and physical-fitness-related outcomes, and to overcome aforementioned context-based challenges for pregnant women (e.g., timing of PA).

The existing learning system in nursing can advance by moving towards an integrative approach that accounts for multiple sources, network collaboration, and continuous investigation of PA. We facilitate the learning processes of healthcare professionals by elaborating on exercise modalities, and commencing a discussion over the components of physical fitness. Acknowledging physical-fitness components could enhance adherence of pregnant women to PA (see e.g., [39]), and empower healthcare professionals who promote PA.

It is evident that research does not focus on different components of PA or components of physical fitness. In line with DiPietro et al. [27] we argue that the future research should focus on providing recommendations tailored for the »peri-pregnancy« period, i.e. before, during, and after a childbirth. Second; whilst the current study adds to tailoring exercise interventions by elaborating exercise modality and physical-fitness components, lack of detailed information not only about pregnant women but also about the pre-pregnancy condition prevents from optimizing interventions for particular cohorts of pregnant women.

Future research should focus on multiple sources to overcome aforementioned challenges that limit the development of tailored evidence-based PA recommendations. Furthermore, we suggest that, in addition to health outcomes, the future research devotes more attention to the role and plausibility of PA for achieving or maintaining physical-fitness components of pregnant women. Such enriched guidelines could enhance adherence and favourable health outcomes of pregnant women, and improve promotion of PA among the healthcare professionals. However, future research should identify appropriate healthcare professionals to distribute PA recommendations (see e.g., [23]), and, in addition, barriers that prevent from effective distribution of physical activity recommendations. Finally, to complement the existing body of the literature with PA recommendations for pregnant women, future research should ensure the scientific nature of such reviews by e.g., addressing the listed limitations, and expanding the data obtained.

The current study reveals a lack of context-dependency, for instance characteristics of pregnant women such as age, previous PA levels, comorbidities, other measures (e.g., BMI), mental well-being, pregnancy status (e.g., early or late pregnancy, health issues during pregnancy, micronutrient levels etc.), non-communicable diseases with viral infections (see e.g., [71, 72]), and other factors such as personal attitude (see e.g., [73]) that could compromise the development and realization of PA. However, the current study also has some methodological limitations in addition to the limited feasibility of data analysis. First, we deliberately examined the more-recent literature in order to get novel and credible selection of evidence. We omitted analysing non-published papers or papers without a free access. Second, a heterogeneity of findings partially prevented from more thorough analyses. Finally, we did not use data processing software, which to some degree reduces the reliability of the qualitative analysis.

## Conclusion with research agenda

The current study demonstrates numerous favourable health outcomes of PA during pregnancy. Recommendations given by practitioners to pregnant women focus on preforming at least 150 min per week moderate-intensity aerobic PA. However, further explanations are not provided. That being said, practitioners can use our systematic literature review to examine favourable maternal and infant health outcomes with a range of exercise modalities (strengthening, balance, stretching, and exercise modalities combined) in addition to aerobic exercise. Furthermore, the practitioners can learn from the current study about the importance of physical-fitness components. As adherence is consistently deemed a critical success factor for PA [74], accounting for the physical-fitness components, i.e., goal-oriented (tailored), in exercise interventions, could remarkably improve the adherence of pregnant women to PA.

## Supplementary Information

**Additional file 1.** Appendix 1.

**Additional file 2.** Appendix 2.

**Additional file 3.** Appendix 3.

## Data Availability

The datasets used and/or analysed during the current study are available from the corresponding author on reasonable request.

## Abbreviations

BMI: Body mass index
GDM: Gestational diabetes mellitus
GRADE: The grading of recommendations, assessment, development, and evaluation system
n: Number of participants
OGTT: Oral glucose tolerance test
p: *p* value of statistical significance
PA: Physical activity
PIO: Population, intervention and outcome
RCT: Randomised controlled trial
SWEP: Study of water exercise during pregnancy

## References

[1] World Health Organization (2022). What does “physical activity” mean?.

[2] Burnett CWF (1956). Value of prenatal exercises. J Obstet Gynaecol.

[3] Simmons D (2017). Effect of physical activity and/or healthy eating on GDM risk: the DALI lifestyle study. J Clin Endocrinol Metab.

[4] Broekhuizen K (2018). Cost-effectiveness of healthy eating and/or physical activity promotion in pregnant women at increased risk of gestational diabetes mellitus: economic evaluation alongside the DALI study, a European multicenter randomized controlled trial. Int J Behav Nutr Phys Act.

[5] Mizgier M (2018). The impact of physical activity during pregnancy on maternal weight and obstetric outcomes. Ginekol Pol.

[6] Kołomańska D, Zarawski M, Mazur-Bialy A (2019). Physical activity and depressive disorders in pregnant women—a systematic review. Medicina.

[7] Shakeel N (2018). Physical activity in pregnancy and postpartum depressive symptoms in a multiethnic cohort. J Affect Disord.

[8] Sun W (2018). Physical activity and body image dissatisfaction among pregnant women: a systematic review and meta-analysis of cohort studies. Eur J Obstet Gynecol Reprod Biol.

[9] Santo EC (2017). Determinants of physical activity frequency and provider advice during pregnancy. BMC Pregnancy Childbirth.

[10] Caspersen CJ, Powell KE, Christenson GM (1985). Physical activity, exercise, and physical fitness: definitions and distinctions for health-related research. Public Health Rep.

[11] Grenier LN (2020). Be healthy in pregnancy: exploring factors that impact pregnant women's nutrition and exercise behaviours. Matern Child Nutr.

[12] Whitaker KM (2016). African American and White women׳s perceptions of weight gain, physical activity, and nutrition during pregnancy. Midwifey.

[13] da Silva GS (2017). A randomized controlled trial of exercise during pregnancy on maternal and neonatal outcomes: results from the PAMELA study. Int J Behav Nutr Phys Act.

[14] O’Brien OA (2017). Influences on the food choices and physical activity behaviours of overweight and obese pregnant women: a qualitative study. Midwifery.

[15] Akbari A (2016). Assessing of physical activity self-efficacy and knowledge about benefits and safety during pregnancy among women. RJMS.

[16] Rodrigues Domingues M, Barros AJD (2007). Leisure-time physical activity during pregnancy in the 2004 Pelotas Birth Cohort Study. Rev Saúde Pública.

[17] Hopkinson Y (2018). Midwives understanding of physical activity guidelines during pregnancy. Midwifery.

[18] Cannon S (2020). A review of pregnancy information on nutrition, physical activity and sleep websites. Women Birth.

[19] Murray DM, Fisher JD (2022). The internet: a virtually untapped tool for research. J Technol Hum Serv.

[20] Mottola MF, Davenport MH, Ruchat SM, Davies GA, Poitras VJ, Gray CE, Jaramillo Garcia A, Barrowman N, Adamo KB, Duggan M, Barakat R, Chilibeck P, Fleming K, Forte M, Korolnek J, Nagpal T, Slater LG, Stirling D, Zehr L (2018). 2019 Canadian guideline for physical activity throughout pregnancy. Br J Sports Med.

[21] May LE (2013). Exercise during pregnancy: the role of obstetric providers. J Osteopath Med.

[22] Hamilton K (2019). Being active in pregnancy: theory-based factors associated with physical activity among pregnant women. Women Health.

[23] De Vivo M, Mills H (2019). “They turn to you first for everything”: insights into midwives’ perspectives of providing physical activity advice and guidance to pregnant women. BMC Pregnancy Childbirth.

[24] Evenson KR, Mottola MF, Artal R (2019). Review of recent physical activity guidelines during pregnancy to facilitate advice by health care providers. Obstet Gynecol Surv.

[25] Lindqvist M, Persson M, Mogren I (2018). “Longing for individual recognition”–pregnant women’s experiences of midwives’ counselling on physical activity during pregnancy. Sex Reprod Healthc.

[26] Lindqvist M, Persson M, Mogren I (2018). “Longing for individual recognition”–pregnant women’s experiences of midwives’ counselling on physical activity during pregnancy. Sex Reprod Healthc.

[27] DiPietro L, Evenson KR, Bloodgood B, Sprow K, Troiano RP, Piercy KL, Vaux-Bjerke A, Powell KE (2019). Benefits of physical activity during pregnancy and postpartum: an umbrella review. Med Sci Sports Exerc.

[28] Gopalakrishnan S, Ganeshkumar P (2013). Systematic reviews and meta-analysis: understanding the best evidence in primary healthcare. J Fam Med Prim Care.

[29] Schardt C (2007). Utilization of the PICO framework to improve searching PubMed for clinical questions. BMC Med Inform Decis Mak.

[30] Hiebl MRW (2021). Sample selection in systematic literature reviews of management research. Organ Res Methods.

[31] Pieper D, Antoine SL, Neugebauer EA, Eikermann M (2014). Up-to-dateness of reviews is often neglected in overviews: a systematic review. J Clin Epidemiol.

[32] Moher D (2009). Preferred reporting items for systematic reviews and meta-analyses: the PRISMA statement. PLoS Med.

[33] Andrews JC (2013). GRADE guidelines: 15. Going from evidence to recommendation—determinants of a recommendation's direction and strength. J Clin Epidemiol.

[34] Balshem H (2011). GRADE guidelines: 3. Rating the quality of evidence. J Clin Epidemiol.

[35] Barakat R (2019). Exercise during pregnancy has a preventative effect on excessive maternal weight gain and gestational diabetes. A randomized controlled trial. Braz J Phys Ther.

[36] Fontana Carvalho AP (2020). Effects of lumbar stabilization and muscular stretching on pain, disabilities, postural control and muscle activation in pregnant woman with low back pain. Eur J Phys Rehabil Med.

[37] de Vargas Nunes Coll C (2019). Efficacy of regular exercise during pregnancy on the prevention of postpartum depression: the PAMELA randomized clinical trial. JAMA Netw Open.

[38] Rodríguez-Blanque R (2020). Water exercise and quality of life in pregnancy: a randomised clinical trial. Int J Environ Res Public Health.

[39] Dodd JM, Deussen AE, Louise J (2019). A randomised trial to optimise gestational weight gain and improve maternal and infant health outcomes through antenatal dietary, lifestyle and exercise advice: the OPTIMISE randomised trial. Nutrients.

[40] Brik M (2019). Does exercise during pregnancy impact on maternal weight gain and fetal cardiac function? A randomized controlled trial. Ultrasound Obstet Gynecol.

[41] Keskin Y (2020). Effectiveness of home exercise in pregnant women with carpal tunnel syndrome: randomized control trial. J Pak Med Assoc.

[42] OʼConnor PJ (2018). Effects of resistance training on fatigue-related domains of quality of life and mood during pregnancy: a randomized trial in pregnant women with increased risk of back pain. Psychosom Med.

[43] Krohn Garnæs K (2019). Effects of supervised exercise training during pregnancy on psychological well-being among overweight and obese women: secondary analyses of the ETIP-trial, a randomised controlled trial. BMJ Open.

[44] Backhausen MG (2017). The effects of an unsupervised water exercise program on low back pain and sick leave among healthy pregnant women: a randomised controlled trial. PLoS ONE.

[45] Gustafsson MK (2019). The effect of an exercise program in pregnancy on vitamin D status among healthy, pregnant Norwegian women: a randomized controlled trial. BMC Pregnancy Childbirth.

[46] Atkinson SA (2022). Be Healthy in Pregnancy (BHIP): a randomized controlled trial of nutrition and exercise intervention from early pregnancy to achieve recommended gestational weight gain. Nutrients.

[47] de Andrade Leão OA, Domingues MR, Bertoldi AD, Ricardo LIC, de Andrade Müller W, Tornquist L, Martins RC, Murray J, Silveira MF, Crochemore-Silva I, Hallal PC, Mielke GI (2022). Effects of regular exercise during pregnancy on early childhood neurodevelopment: the physical activity for mothers enrolled in longitudinal analysis randomized controlled trial. J Phys Act Health.

[48] Gonzalez-Plaza E, Bellart J, Arranz Á, Luján-Barroso L, Mirasol EC, Seguranyes G (2022). Effectiveness of a step counter smartband and midwife counseling intervention on gestational weight gain and physical activity in pregnant women with obesity (Pas and Pes study): randomized controlled trial. JMIR Mhealth Uhealth.

[49] Talebi E, Mohaddesi H, Vahabzadeh D (2022). Examination of influence of social media education through mobile phones on the change in physical activity and sedentary behavior in pregnant women: a randomized controlled trial. BMC Womens Health.

[50] Sandborg J (2022). HealthyMoms : a smartphone application to promote healthy weight gain, diet and physical activity during pregnancy : a randomized controlled trial.

[51] Niaraki MR, Pakniat H, Alizadeh A, Hosseini MA, Ranjkesh F (2021). Effect of exercise in water on the musculoskeletal pain in pregnant women: a randomized controlled trial. J Musculoskelet Res.

[52] Hayden JA (2021). Some types of exercise are more effective than others in people with chronic low back pain: a network meta-analysis. J Physiother.

[53] Shimada IS (2019). Effects of exercise therapy on painful temporomandibular disorders. J Oral Rehabil.

[54] Murphy SE (2022). Influence of exercise type on maternal blood pressure adaptation throughout pregnancy. AJOG Glob Rep.

[55] Haskell WL, Montoye HJ, Orenstein D (1985). Physical activity and exercise to achieve health-related physical fitness components. Public Health Rep.

[56] Embaby H, Elsayed E, Fawzy M (2016). Insulin sensitivity and plasma glucose response to aerobic exercise in pregnant women at risk for gestational diabetes mellitus. Ethiop J Health Sci.

[57] Flynn AC, Seed PT, Patel N, Barr S, Bell R, Briley AL, Godfrey KM, Nelson SM, Oteng-Ntim E, Robinson SM, Sanders TA, Sattar N, Wardle J, Poston L, Goff LM, UPBEAT consortium (2016). Dietary patterns in obese pregnant women; influence of a behavioral intervention of diet and physical activity in the UPBEAT randomized controlled trial. Int J Behav Nutr Phys Act.

[58] Kirkham AA (2018). Exercise prescription and adherence for breast cancer: one size does not FITT all. Med Sci Sports Exerc.

[59] Reid RER, Thivel D, Mathieu M-E (2019). Understanding the potential contribution of a third “T” to FITT exercise prescription: the case of timing in exercise for obesity and cardiometabolic management in children. Appl Physiol Nutr Metab.

[60] Burnet K (2019). How fitting is FIIT? A perspective on a transition from the sole use if frequency, intensity, time, and type in exercise prescription. Psychol Behav.

[61] Malta MB (2016). Educational intervention regarding diet and physical activity for pregnant women: changes in knowledge and practices among health professionals. BMC Pregnancy Childbirth.

[62] De Vivo M, Mills H (2021). Laying the foundation for pregnancy physical activity profiling: a framework for providing tailored physical activity advice and guidance to pregnant women. Int J Environ Res Public Health.

[63] Pate RR (1988). The evolving definition of physical fitness. Quest.

[64] Campbell N, De Jesus S, Prapavessis H, Gellman ME, Turner JR (2013). Physical fitness. Encyclopedia of behavioral medicine.

[65] Marini S (2021). Co-design and evaluation of the feasibility and the efficacy of a multiple-targeted adapted physical activity intervention to promote quality of life, well-being and physical activity levels in pregnant women: the “WELL-DONE!” study protocol. Sustainability.

[66] Marín-Jiménez N (2019). Association of self-reported physical fitness with pain during pregnancy: the GESTAFIT project. Scand J Med Sci Sports.

[67] O’Connor PJ (2011). Safety and efficacy of supervised strength training adopted in pregnancy. J Phys Act Health.

[68] White E, Pivarnik J, Pfeiffer K (2014). Resistance training during pregnancy and perinatal outcomes. J Phys Act Health.

[69] Henriksson P (2021). Associations of body composition and physical fitness with gestational diabetes and cardiovascular health in pregnancy: results from the HealthyMoms trial. Nutr Diabetes.

[70] Figueira HA (2014). Pregnancy-related low back pain relief after maximum static flexibility program. Health.

[71] Zovko V, Budler M (2021). Sitting ducks: physical activity and diet-related interventions in the" peri-COVID-19" period. Kinesiol Slov.

[72] Sitzberger C (2022). Physical activity in high-risk pregnancies. J Clin Med.

[73] Hamilton K (2019). Being active in pregnancy: theory-based factors associated with physical activity among pregnant women. Women Health.

[74] Room J (2017). What interventions are used to improve exercise adherence in older people and what behavioural techniques are they based on? A systematic review. BMJ Open.

