# Supplementary material for: Physical activity during pregnancy: a systematic review for the assessment of current evidence with future recommendations
Source: BMC Sports Sci Med Rehabil. 2022 Jul 16;14:133. doi: 10.1186/s13102-022-00524-z (PMC9288689; doi:10.1186/s13102-022-00524-z)
Supplement: Supplementary file 2 — Additional file 2. Appendix 2. [file 13102_2022_524_MOESM2_ESM.docx]

Additional file 2: Appendix 1. Excluded studies with exclusion reason

| **Reference** | **Exclusion reason** |
| --- | --- |
| Sklempe Kokic, I., Ivanisevic, M., Uremovic, M., Kokic, T., Pisot, R. & Simunic, B. (2017). Effect of therapeutic exercises on pregnancy-related low back pain and pelvic girdle pain: Secondary analysis of a randomized controlled trial. *J Rehabil Med*, 49(3), pp. 251-257. doi: 10.2340/16501977-2196. | Study type (a secondary analysis) |
| Bowman, E.N., Elshaar, R., Milligan, H., Jue, G., Mohr, K., Brown, P., Watanabe, D.M. & Limpisvasti, O. (2019). Proximal, Distal, and Contralateral Effects of Blood Flow Restriction Training on the Lower Extremities: A Randomized Controlled Trial. *Sports Healt*, 11(2), pp. 149-156. doi: 10.1177/1941738118821929. | Sample |
| Broberg, L., Backhausen, M., Damm, P., Bech, P., Tabor, A. & Hegaard, H.K. (2017). Effect of supervised exercise in groups on psychological well-being among pregnant women at risk of depression (the EWE Study): study protocol for a randomized controlled trial. *Trials,* 18(1), p. 210. doi: 10.1186/s13063-017-1938-z. | Study type (protocol - no results) |
| Antoun, E., Kitaba, N.T., Titcombe, P., Dalrymple, K.V., Garratt, E.S., Barton, S.J., Murray, R., Seed, P.T., Holbrook, J.D., Kobor, M.S., Lin, D.T., MacIsaac, J.L., Burdge, G.C., White, S.L., Poston, L., Godfrey, K.M. & Lillycrop, K.A.; UPBEAT Consortium. (2020). Maternal dysglycaemia, changes in the infant's epigenome modified with a diet and physical activity intervention in pregnancy: Secondary analysis of a randomised control trial. *PLoS Med,* 17(11), p. e1003229. doi: 10.1371/journal.pmed.1003229. | Study type (a secondary analysis) |
| Baena-García, L., Coll-Risco, I., Ocón-Hernández, O., Romero-Gallardo, L., Acosta-Manzano, P., May, L. & Aparicio, V.A. (2020). Association of objectively measured physical fitness during pregnancy with maternal and neonatal outcomes. The GESTAFIT Project. *PLoS One,* 15(2), p. e0229079. doi: 10.1371/journal.pone.0229079. | Study type (longitudinal study) |
| Haakstad, L. A., Vistad, I., Sagedal, L. R., Lohne-Seiler, H. & Torstveit, M. K. (2018). How does a lifestyle intervention during pregnancy influence perceived barriers to leisure-time physical activity? The Norwegian fit for delivery study, a randomized controlled trial. *BMC pregnancy and childbirth*, 18(1), pp. 1-10. Doi: https://doi.org/10.1186/s12884-018-1771-8 | Study type (a secondary analysis) |
| McDonald, S. M., Yeo, S., Liu, J., Wilcox, S., Sui, X. & Pate, R. R. (2020). Association between change in maternal physical activity during pregnancy and infant size, in a sample overweight or obese women. *Women & Health*, 60(8), pp. 929-938. Doi: https://doi.org/10.1080/03630242.2020.1779904 | Study type (a secondary analysis) |
| Haakstad, L. A., Sanda, B., Vistad, I., Sagedal, L. R., Seiler, H. L. & Torstveit, M. K. (2017). Evaluation of implementing a community-based exercise intervention during pregnancy. *Midwifery*, 46, pp. 45-51. Doi: https://doi.org/10.1016/j.midw.2017.01.010 | Study type (a descriptive study) |
