# Supplementary material for: Physical activity during pregnancy: a systematic review for the assessment of current evidence with future recommendations
Source: BMC Sports Sci Med Rehabil. 2022 Jul 16;14:133. doi: 10.1186/s13102-022-00524-z (PMC9288689; doi:10.1186/s13102-022-00524-z)
Supplement: Supplementary file 3 — Additional file 3. Appendix 3. [file 13102_2022_524_MOESM3_ESM.docx]

Additional file 3: Appendix 2. Quality assessment of included studies

| **No.** | **Study design** | **Study limitations** | **Inconsistency** | **Indirectness** | **Imprecision** | **Risk of bias** | **GRADE** |
| --- | --- | --- | --- | --- | --- | --- | --- |
|  | RCT | - Nutrition and energy intake was not monitored; - Differences in participants educational levels; - Supervised activity program on a mass scale; - Only Spanish population; two tertiary care hospitals. | Higher deviations in CIs for maternal and infant outcomes. | No serious indirectness. | No serious imprecision. | No serious risk of bias. | ++ |
|  | RCT | - The absence of the control group; - EMG measurements for standing position were not performed; - Small sample size; - Absence of follow-up. | Higher deviations in CIs for both interventions. | No serious indirectness. | No serious imprecision. | No serious risk of bias. | ++ |
|  | RCT | - Lack of significant effect of the intervention; - Exclusion criteria; - Lack of clinical diagnosis of depression; - Depressive symptoms were not accessed at the baseline. | No serious inconsistency. | No serious indirectness. | No serious imprecision. | No serious risk of bias. | +++ |
|  | RCT | - Small sample size; - Exclusion criteria. | No serious inconsistency. | No serious indirectness. | No serious imprecision. | No serious risk of bias. | +++ |
|  | RCT | - Questionable internal consistency of the questionnaire; - Small sample size; - Authors did not report all study limitations. | CIs are not reported. | No serious indirectness. | No serious imprecision. | Moderate risk of bias. | + |
|  | RCT | - Lack of statistical power and low compliance; - Authors did not report all study limitations. | No serious inconsistency. | No serious indirectness. | No serious imprecision. | Moderate risk of bias. | + |
|  | RCT | - Authors did not report all study limitations. | Higher deviations in CIs for both infant and maternal outcomes. | No serious indirectness. | No serious imprecision. | Moderate risk of bias. | + |
|  | RCT | - Limited sample size; - Size differences between two groups; - Potential sample selection bias; - No follow-up. | CIs are not reported. | No serious indirectness. | No serious imprecision. | No serious risk of bias. | ++ |
|  | RCT | - Relatively small sample size; - Authors did not report all study limitations. | CIs are not reported. | No serious indirectness. | No serious imprecision. | Moderate risk of bias. | + |
|  | RCT | - Relatively small sample size; - Authors did not report all study limitations. | CIs are not reported. | No serious indirectness. | No serious imprecision. | Moderate risk of bias. | + |
|  | RCT | - Small sample size; - High percentage of those who dropped out; - Low adherence to the exercise protocol. | Higher deviations in CIs for psychological well-being and anxiety. | No serious indirectness. | No serious imprecision. | No serious risk of bias. | ++ |
|  | RCT | - Small effects of specific interventions; - Sample consisted of relatively healthy participants (not representative); - Lack of blinding; - Lack of robust analyses. | Higher deviations in CIs for low back pain. | No serious indirectness. | No serious imprecision. | No serious risk of bias. | ++ |
|  | RCT | - Self-reporting; - Recall bias; - Limited availability of unit costs; - Much missing data. | Higher deviations in CIs in cost effectiveness analyses. | No serious indirectness. | No serious imprecision. | No serious risk of bias. | ++ |
|  | RCT | - Questionable sample representability; - Loss to follow-up; - Lack of adherence in intervention group. | CIs are not reported. | No serious indirectness. | No serious imprecision. | No serious risk of bias. | ++ |
|  | RCT | - Lack of generalizability; lack in sample characteristics; relatively small sample size. | No serious inconsistency. | No serious indirectness. | No serious imprecision. | No serious risk of bias. | ++ |
|  | RCT | - Large drop-outs; low adherence; lack of control; use of different neurodevelopment measures. | No serious inconsistency. | No serious indirectness. | No serious imprecision. | No serious risk of bias. | ++ |
|  | RCT | - Small sample size; large drop-outs; lack of analyses; all factors were not measured and analysed. | No serious inconsistency. | No serious indirectness. | No serious imprecision. | No serious risk of bias. | ++ |
|  | RCT | - Small sample size; lack of control; lack of access to smartphone. | CIs are not reported. | No serious indirectness. | No serious imprecision. | No serious risk of bias. | + |
|  | RCT | - Small sample size; lack of generalizability; risk of recall bias. | No serious inconsistency. | No serious indirectness. | No serious imprecision. | No serious risk of bias. | ++ |
|  | RCT | - Small sample size; lack in comparison group; lack of generalizability; costs. | No serious inconsistency. | No serious indirectness. | No serious imprecision. | No serious risk of bias. | ++ |

*Final GRADE ranking: ++++ high quality: +++ moderate; ++ low; + very low*

*EMG = electromyography; RCT = randomized controlled trial; CI = confidence interval*
